# Microstructure and Mechanical Properties of Zn-Ni-Al_2_O_3_ Composite Coatings

**DOI:** 10.3390/ma11050853

**Published:** 2018-05-21

**Authors:** Yang Bai, Zhenhua Wang, Xiangbo Li, Guosheng Huang, Caixia Li, Yan Li

**Affiliations:** 1 College of Mechanical and Electrical Engineering, China University of Petroleum (East China), Qingdao 266580, China; b14040127@s.upc.edu.cn; 2 Science and Technology on Marine Corrosion and Protection Laboratory, Luoyang Ship Materials Research Institute, Qingdao 266237, China; wzh_dut@163.com (Z.W.); lixb@163.com (X.L.); huanggs@sunrui.net (G.H.); 15054242571@163.com (C.L.)

**Keywords:** low-pressure cold spray, Zn-Ni composite coating, microstructure, wear resistance

## Abstract

Zn-Ni-Al_2_O_3_ composite coatings with different Ni contents were fabricated by low-pressure cold spray (LPCS) technology. The effects of the Ni content on the microstructural and mechanical properties of the coatings were investigated. According to X-ray diffraction patterns, the composite coatings were primarily composed of metallic-phase Zn and Ni and ceramic-phase Al_2_O_3_. The energy-dispersive spectroscopy results show that the Al_2_O_3_ content of the composite coatings gradually decreased with increasing of Ni content. The cross-sectional morphology revealed thick, dense coatings with a wave-like stacking structure. The process of depositing Zn and Ni particles and Al_2_O_3_ particles by the LPCS method was examined, and the deposition mechanism was demonstrated to be mechanical interlocking. The bond strength, micro hardness and friction coefficient of the coatings did not obviously change when the Ni content varied. The presence of Al_2_O_3_ and Ni increased the wear resistance of the composite coatings, which was higher than that of pure Zn coatings, and the wear mechanism was abrasive and adhesive wear.

## 1. Introduction

Zn-Ni alloy coatings have been widely used in a range of industrial applications due to their superior corrosion resistance [1]. These coatings are employed as sacrificial coatings for steel because they preferentially dissolve in corrosive media and result in a surface layer of products with low solubility that slows the corrosion reaction and protects the substrate underneath. Many published investigations have reported that the corrosion resistance of Zn-Ni alloy coating, therein indicating the corrosion resistance of such coatings being several times higher than that of pure Zn coatings because the Ni addition [−0.257 V vs. the standard hydrogen electrode (SHE)] can reduce the potential difference between Zn (−0.762 V vs. SHE) and Fe (−0.441 V vs. SHE) and the galvanic cell electromotive force [2,3,4,5,6]. However, research on the wear resistance of Zn-Ni alloy coatings is limited, though improving the wear resistance of Zn-Ni alloy coatings by adding reinforcements such as carbide and ceramic particles (Al_2_O_3_, SiC, WC, CNTs, etc.) has been investigated [7,8,9]. The Zn-Ni-Al_2_O_3_ composite coatings obtained by this method have been considered to have excellent weld ability, high temperature resistance, formability, nearly complete resistance to hydrogen embrittlement and other advantageous characteristics [10,11,12,13,14].

Generally, Zn-Ni composite coatings are prepared on the surface of materials and components using electroplating [8,15], hot dip coating [16,17] and thermal spraying methods [18,19,20,21]. Thermal spraying technology is more cost-effective and environment-friendly and can improve the thickness of coatings, which is necessary for long-term protection [19,20,22,23]. However, thermally sprayed materials may undergo certain microstructural changes, oxidation and/or grain growth, leading to highly porous coatings, high oxygen contents, heat stress and other defects [24,25]. Therefore, a new spray technology for high-performance coatings is sought.

In recent years, cold spray (CS) technology, with its several unique characteristics, has emerged in the field of surface engineering. CS is a solid-state material deposition technique, in which micrometer-sized feedstock bond to a substrate via high-velocity impact, with the associated severe plastic deformation [25,26,27]. In this process, the feedstock is accelerated to supersonic speeds (300–1200 m/s) within a converging-diverging nozzle using a carrier gas [28,29]. Once the particles reach a critical velocity upon impact against a surface, they deform sufficiently to create a dense coating [30]. This process involves no heating above the melting temperature during coating deposition; thus, oxidation, grain growth, residual tensile stress, phase changes and other unwanted chemical reactions associated with thermal spray methods can be avoided [31,32]. A wide range of materials, such as metals, alloys, intermetallics and composites, can be successfully deposited by CS technology.

Two categories of CS systems, namely the high- and low-pressure systems, currently exist [33]. High-and low-pressure systems attain gas pressure ranges of 2–5 MPa, and 0.3–1 MPa, respectively. High-pressure systems can achieve a higher particle velocity (1000 m·s^−1^) than the low-pressure systems, which in turn provides higher deposition efficiency. The major drawback of these systems is their high operating costs due to being operated under N_2_, He or other inert gases by a corresponding spraying device. The low-pressure cold spray (LPCS) technique has been widely implemented due to its outstanding advantages, including its simple and portable equipment and its ability to improve the performance of coatings, which exhibit large thicknesses, good adhesion and low porosities [34,35,36,37].

However, the significant difference between the melting points of Zn and Ni complicates the alloying process. Therefore, preparing a Zn-Ni alloy coating with a high Ni content is challenging. Present research on the preparation of Zn-Ni-Al_2_O_3_ composite coatings by LPCS is limited, and the tribological properties of LPCS Zn-Ni-Al_2_O_3_ coating are even less extensively explored. In this study, LPCS was used to produce Zn-Ni-Al_2_O_3_ composite coatings. The aim of this study is to investigate the microstructure, mechanical properties and tribological behavior of these coatings. In addition, the effects of the Ni concentration on the properties of the coating are discussed.

## 2. Materials and Methods

### 2.1. Materials and Coating Technique

**Figure 1:**
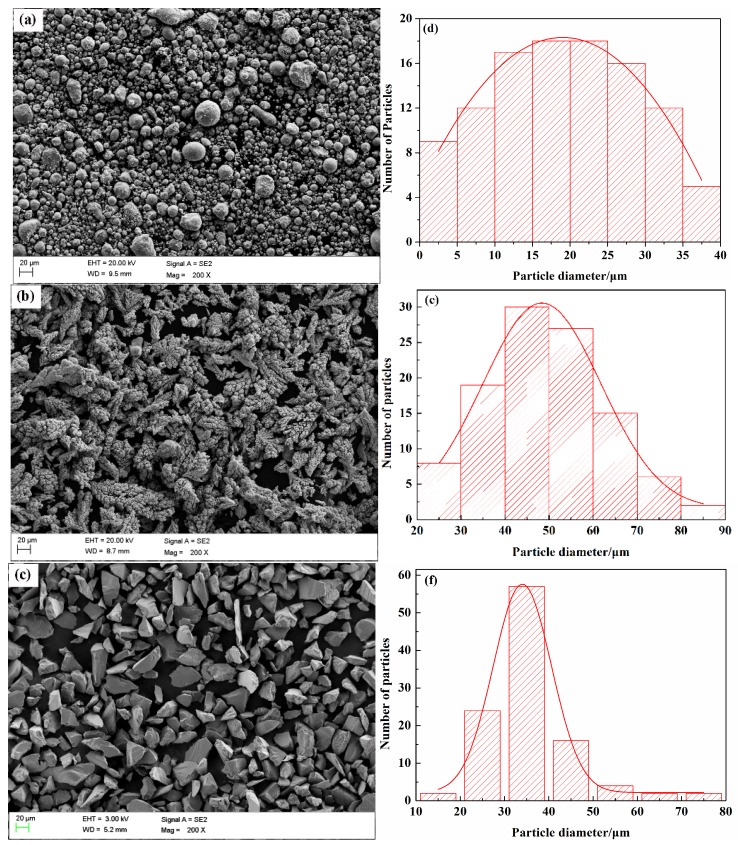
SEM images of the spraying powders: (**a**) Zn, (**b**) Ni and (**c**) alumina and particle size analysis of powders: (**d**) Zn, (**e**) Ni and (**f**) alumina.

Commercially available spraying powders of Zn (Xinri Zinky Industry Co. Ltd., Shijiazhuang, China), electrolytic Ni (Shanghai Jiujia Pulvenous Material Co. Ltd., Shanghai, China) and alumina (Precursor Plasma Powders Co. Ltd., Yiyang, China) were used in the spraying process. The morphologies of the selected powders are shown in Figure 1a–c. The particle size distributions of at least 100 particles were measured and manually counted using ImageJ software (version 2X, National Institutes of Health, Bethesda, MD, USA). The results were transferred to origin-8.5 (Origin Lab Corporation, Northampton, MA, USA), where a histogram chart was prepared. This technique of particle size analysis is aligned with reported procedures [38]. The size distribution of powders is shown in Figure 1d–f. The Zn powders were produced by the atomizing method, and the powder size distribution varied from 2 to 40 µm with a purity higher than 96% (Figure 1a,d). Figure 1b,e show that the size of the Ni particles was in the range of +20–90 µm with a purity higher than 99.5%. The Ni powders were produced by the electrolytic method, and the particles were dendritic. The alumina powders had a particle size range of +15–80 µm with a purity higher than 99.5%. The powders exhibited irregular edges, as shown in Figure 1c,f, and they were produced by settlement encapsulation–dehydration.

### 2.2. Experimental Procedure and Characterization Techniques

The Zn-Ni-Al_2_O_3_ spraying powders were mechanically mixed from Zn and electrolytic Ni powders at designed ratios (1:9, 3:17, 1:4, 1:3) and then separately blended with 30 vol % alumina powders. This given reinforcement volume fraction (30 vol % Al_2_O_3_) was obtained from the literature research and the preliminary experiments. According to the literature, a typical range of the composition of Al_2_O_3_ in the feedstock powder is 25–50 vol % [39,40]. On this basis, pre-experiments were carried out and found that the deposition efficiency of the coating with 30 vol % Al_2_O_3_ is higher than that of the coatings with 40 and 50 vol % Al_2_O_3_, and the thickness of this coating can meet our requirements. This is why we considered this composition. The substrate material was Q235 carbon steel with the following chemical composition (wt %): 0.49% C, 0.37% Si, 0.40% Mn and Fe balance. The dimensions of the substrate coupons were 100 × 50 × 3.5 mm^3^. Before spraying, the substrates were degreased ultrasonically in acetone and grit-blasted with corundum (particle size of 2 mm) to remove any contamination from the surface. The composite coatings were sprayed using an LPCS instrument (DYMET 413, Obninsk, Russia) with the process parameters specified in Table 1. Compressed air was used as the working gas, with a driving pressure of 0.60 MPa and a gas temperature of 400 °C. The standoff distance from the gun exit to the substrate surface was 25 mm. The nozzle traverse speed and the powder feed rate were set to 20 mm/s and 12 g/min, respectively. The thickness of the composite coatings was measured with a 3D digital microscope system (HIROX KH-8700, Tokyo, Japan), and the thickness of LPCS Zn-Ni-Al_2_O_3_ composite coatings with Ni powder contents of 10, 15, 20 and 25 wt % was 276 ± 13, 285 ± 15, 290 ± 22, and 313 ± 17 μm, respectively.

The morphologies and elemental distributions of the coatings were examined by scanning electron microscopy (SEM, Carl Zeiss Ultra 55, Braunschweig, Germany) coupled with energy-dispersive spectroscopy (EDS, Oxford X-Max 50, High Wycombe, UK). The coated samples (10 × 10 mm^2^) were prepared by first grinding them first with SiC papers, subsequent polishing with diamond slurries and colloidal silica, degreasing with alcohol, and drying with a hair dryer.

X-ray diffraction (XRD, D8 Advance, Bruker, Bremen, Germany) analyses of the coatings were performed with Co Kαradiation with a wavelength of 0.17902 nm, scanning angle range of 5–90°, scan step size of 0.02°/s, and scanning speed of 3.8636°/min.

The roughnesses of the composite coatings was measured using a 3D digital microscope system (HIROX KH-8700, Tokyo, Japan), and the average of 5 measurements was used as the roughness value.

The Vickers microhardness was measured on the cross-section of the coatings using a digital microhardness tester (HVS-1000, Shanghai, China) with a load of 300 g and a holding time of 15 s. To reduce systematic error, for each sample, ten indentations were measured on the polished cross-section and the average microhardness value was determined.

The specimens were bonded to 304 L stainless steel cylinders (Ø 25 mm × 5 mm) with E-7 adhesive (epoxy resin and curing agent, ratio of 9:1) and then cured at 100 °C for 3 h. The adhesion strength of the tensile specimens (Ø 25 mm × 3 mm) was measured using a constant-load pre-stress corrosion cracking test (SANS CMT 5305, Shanghai, China) with a crosshead speed of 0.03 mm/s. Three specimens were tested for each experimental run and the adhesion strength was given as an average of 3 measurements.

The friction coefficients were measured with a friction and wear tester (CETR UMT-3, Campbell, CA, USA) and the linear ball-on-flat reciprocating sliding method. The friction coefficient was obtained with a load of 5 N and speed of 3 mm/s; the wear loss was examined through the movement of a chrome steel ball (Ø 4 mm) surface with a load of 5 N, a sliding speed of 3 mm/s and a track length of 10 mm. A wear test was run to 10,000 sliding cycles, corresponding to total sliding distance of 100 m. The wear loss weight of the specimens was evaluated using an electronic analytical balance.

## 3. Results and Discussion

### 3.1. Microstructural Characterization of the Composite Coatings

**Figure 2:**
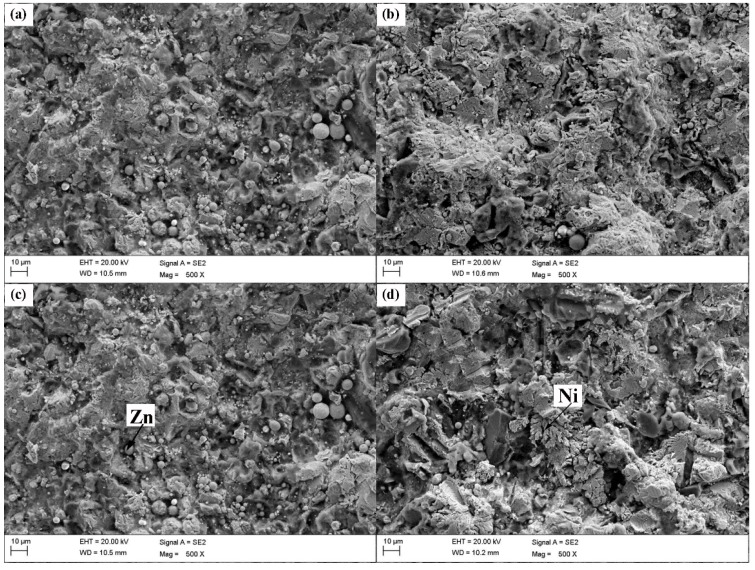
Scanning electron micrographs of the composite coatings with different amounts of Ni powders: (**a**) 10 wt %, (**b**) 15 wt %, (**c**) 20 wt %, and (**d**) 25 wt %. Arrows indicate intact spherical Zn particles and dendritic Ni particles.

**Figure 3:**
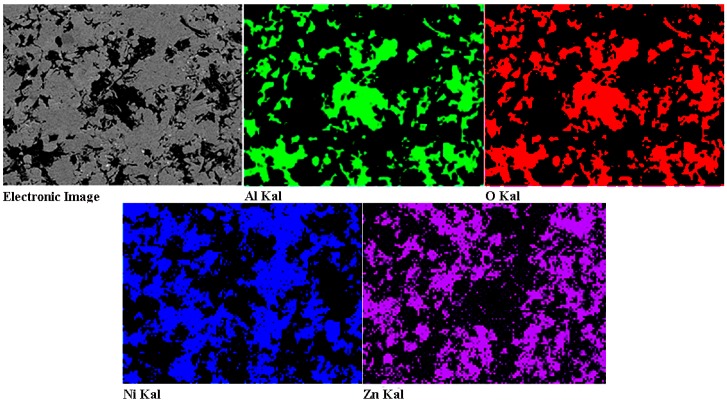
EDS mapping analysisof the polished top surface of (80 wt % Zn–20 wt % Ni)-30 vol % Al_2_O_3_ composite coatings.

The SEM photographs in Figure 2 show the surface morphologies of the four types of Zn-Ni-Al_2_O_3_ composite coatings. The surfaces of the composite coatings exhibit many shallow pits from being hit by successive particles and a small number of inadequate, deformed particles. The roughnesses of the composite coatings with Ni powder contents of 10, 15, 20 and 25 wt % are 0.5600 ± 0.0386, 0.5704 ± 0.0431, 0.5882 ± 0.0358, and 0.5783 ± 0.0371 μm, respectively. The roughnesses of coatings slightly change with an increasing Ni content, mainly due to the influence of the coating thickness. Note that some researchers have discovered that the roughness of the coating surface increases linearly with the thickness of the coating [41,42]. Figure 3 shows the EDS mapping analysis of the polished top surface of the (80 wt % Zn–20 wt % Ni)-30 vol % Al_2_O_3_ composite coating, wherein the dark areas are the embedded Al_2_O_3_ ceramic particles, and Zn/Ni compositions are uniformly distributed. The distributions of O and Al shown in the figure are essentially coincident, which indicates that LPCS is a solid-state powder deposition process and that the sprayed particles do not undergo oxidation, phase transition or other undesirable processes. Therefore, the deposited coating not only retains the characteristics of the original materials but also exhibits a compact structure, low porosity and other characteristics that help maintain a high coating quality.

**Figure 4:**
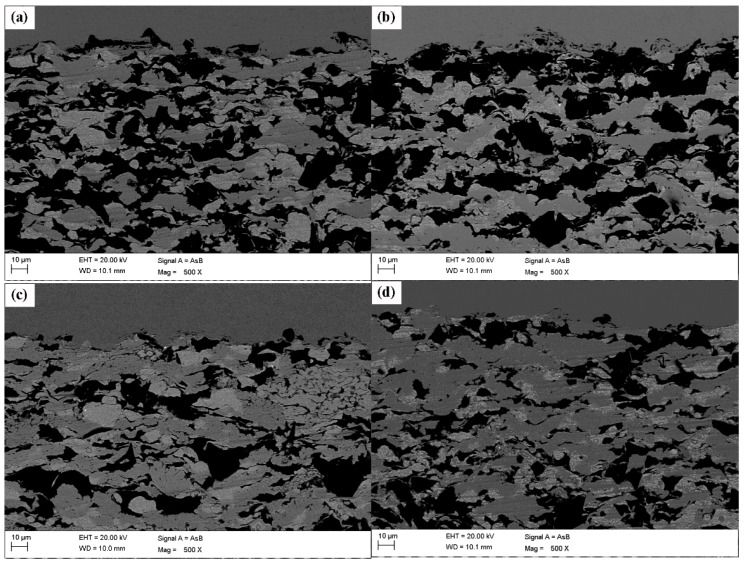
SEM image of the cross section of the composite coatings with different Ni powder contents: (**a**) 10 wt %, (**b**) 15 wt %, (**c**) 20 wt % and (**d**) 25 wt %. The darkest areas are the irregular alumina ceramic, the dark-gray areas are Ni, and light-gray areas are Zn.

**Figure 5:**
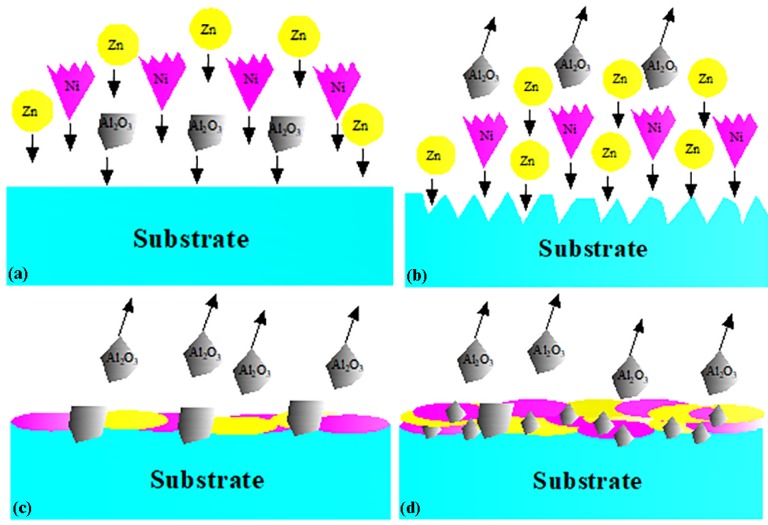
Deposition process of metal (Zn, Ni particles) and ceramic (Al_2_O_3_ particles) with LPCS technology: (**a**) the deposition sequence of the particles prior to deposition, (**b**) the amelioration of the surface profile, (**c**) coating growth by the plastic deformation of Zn and Ni particles and embedding of Al_2_O_3_ particles, and (**d**) the fragmentation of the already-deposited Al_2_O_3_ particles due to the high-velocity impact of the incoming ceramic particles.

The cross-sectional morphology of the composite coatings with different Ni powder contents is shown in Figure 4. The coatings area pseudo-layered structure with a wave-like pattern, and the Al_2_O_3_ ceramic particles are embedded and distributed in the coatings due to the continuous high-velocity impact. The Al_2_O_3_ ceramic particles play several important roles in the activating behavior of the coatings and improving the deposition efficiency of Zn and Ni powders [35,43]. Based on reports in the literature and our experimental results, we propose that the deposition process of metal (Zn, Ni) and ceramic (Al_2_O_3_) particles primarily consists of the following two processes (Figure 5): first, the high-speed impact of metal and ceramic particles changes the surface profile of the substrate, as in a well-known grit-blasting procedure, especially due to the ceramic particles, which improves the bonding between the first coating layer and the substrate; the growth of the deposited coating primarily depends on the plastic deformation of the Zn and Ni particles arising from the high-speed impact of metal particles on the substrate. Second, after the deposition of the first layer, the high-speed impact of Zn, Ni and Al_2_O_3_ particles disrupts the oxide films on the surface of the metal particles and improves the conditions for metallic bonding, creating more favorable conditions for the mechanical interlocking [44] of the particles forming consecutive layers; during this process, the pinning effect produced by the impact of the incoming ceramic particles on the already-deposited metal and ceramic particles leads to the fragmentation and refinement of the already-deposited ceramic particles.

### 3.2. Compositional Analysis of the Composite Coatings

**Figure 6:**
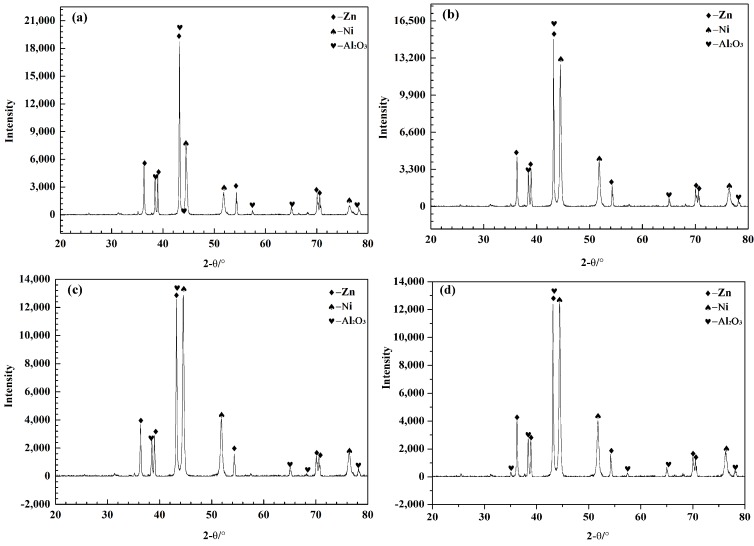
XRD patterns of the composite coatings with different Ni powder contents (**a**) 10 wt %, (**b**) 15 wt %, (**c**) 20 wt % and (**d**) 25 wt %.

Figure 6 shows the XRD patterns of composite coatings with different Ni powder contents: 10, 15, 20 and 25 wt %. The composite coatings are primarily composed of metallic-phase Zn and Ni and ceramic-phase Al_2_O_3_. The coatings exhibit strong diffraction peaks that indicate the presence of elemental Zn and Ni and weak diffraction peaks that indicate the presence of Al_2_O_3_. The absence of other impurity peaks indicates that the coatings do not exhibit new materials or oxidation. As the Ni content increases, the diffraction peaks of elemental Ni obviously strengthen. However, the Al_2_O_3_ content of the composite coatings gradually decreases with increased Ni content, as listed in Table 2. Ni is softer than the Al_2_O_3_ ceramic particles and harder than Zn. Instead of the Al_2_O_3_ particles being embedded in the Zn, an increasing number of Ni particles are embedded into the softer Zn, which scatter some of the Al_2_O_3_ particles into the air, eventually decreasing the Al_2_O_3_ content. Table 2 also shows that, although the Zn:Ni mass ratio in the composite coating is different from that of the mixed powders before spraying, the Ni content in the coating significantly increases when the Ni powder content is increased, indicating that Ni powder with Zn powder is easily deposited when the mass ratio of the Zn:Ni mixed powder is less than or equal to 1:4.

### 3.3. Bond Strength and Microhardness of the Composite Coatings

**Figure 7:**
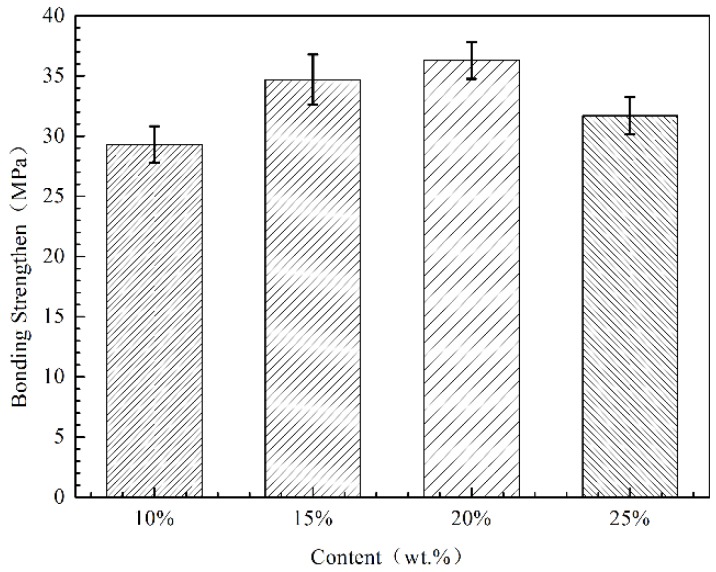
Influence of Ni content on the adhesion strength of coatings.

The bond strength test results are shown in Figure 7. The average bond strength of the four coatings does not linearly increase with variable Ni content. Koivuluoto [45] prepared Zn and Ni coatings using the LPCS technology on steel substrates and obtained bond strengths of 33 and 8 MPa, respectively, due to the small plastic deformation of Ni particles during the spraying process, indicating that excessive Ni decreases the bonding strength of the coating.

**Figure 8:**
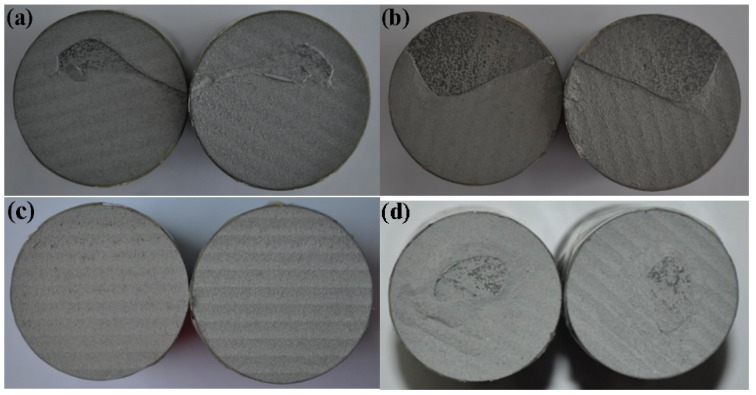
Photograph of the fracture section of the composite coatings with different amounts of Ni powder: (**a**) 10 wt %, (**b**) 15 wt %, (**c**) 20 wt % and (**d**) 25 wt %.

Figure 8 shows the tensile fractures of low-pressure cold-sprayed Zn-Ni composite coatings after pull-off tests. From the pull test section morphology, all coating test fracture surfaces occur between the coating and the substrate. This shows that the cohesion of the coating is higher than that of the coating/matrix. During the CS technique, the bond strength between particles and matrix is related to the hardness difference between the particles and the matrix. The hardness of the steel matrix is much higher than that of the Zn alloy. When the Zn alloy collides with the steel substrate, the deformation of Zn is much higher than that of the matrix. When the Zn particles collide with the surface of the Zn coating, the deformation of the zinc particles is equivalent to the deformation of the coating, resulting in a more compact combination. Thus, the bond strength is much higher than the bond between the steel substrate. The bond strength of the coating therefore depends mainly on the Zn alloy deposition behavior.

**Figure 9:**
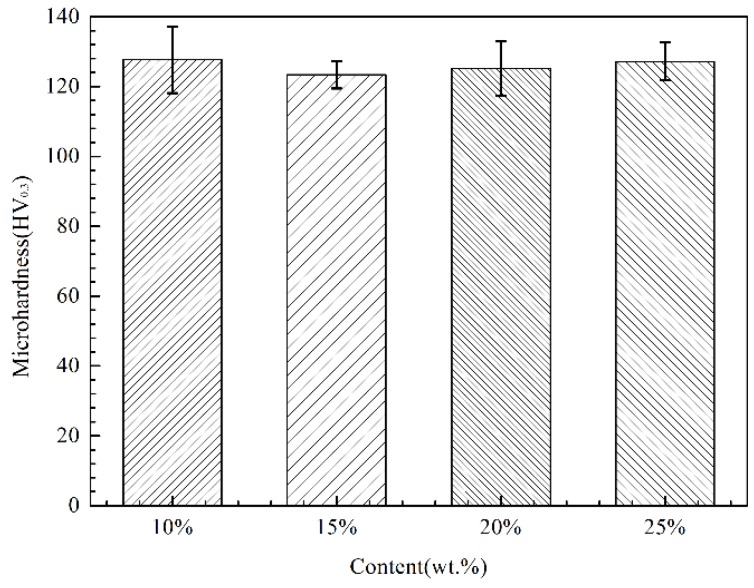
Effect of Ni content on the microhardness of the composite coatings.

Figure 9 shows the Vickers microhardness results of the four different coatings as measured by a digital microhardness tester using the following formula:

HV = 18,169 × P/d^2^ (1)

where P is the applied load in grams and d is the length of the indentation diagonal in millimeters. The microhardness of Zn-Ni composite coatings does not significantly change with the Ni content, and the average microhardness of the coatings is above 120 HV_0.3_. The hardness values of the Zn-Ni-Al_2_O_3_ composite coatings with different Ni contents are greatly improved over the LPCS pure Zn coating (plus 50 vol % of Al_2_O_3_). In this study, the microhardness of the Zn-Ni-Al_2_O_3_ composite coatings is essentially equivalent to that of the Ni coating in the study of Koivuluoto [45], which is mainly because the hardness of the coating is determined by the composition of the zinc, nickel and alumina. The Ni framework is sufficient to support the overall hardness of the coating and therefore exhibits a much higher hardness than pure Zn coating (about 45 HV_0.3_).

### 3.4. Friction and Wear Properties of the Composite Coatings

**Figure 10:**
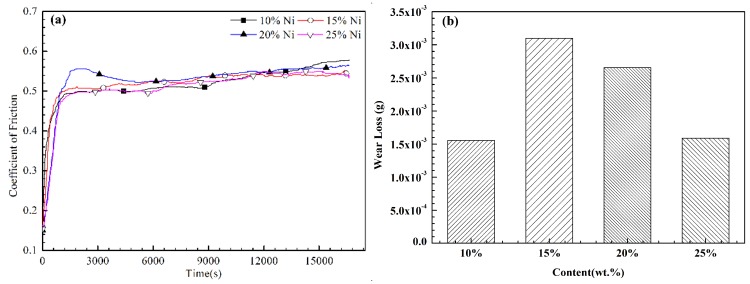
Influence of Ni content on (**a**) coefficient of friction and (**b**) wear lossof the coatings.

Figure 10 shows the friction coefficient (COF) and wear loss of the Zn-Ni composite coatings. Figure 10a shows that, during the initial cycles, the friction coefficient of the coating increases rapidly, and the initially rough coating becomes smooth due to the plowing of the asperities during the running-in stage [46,47]. After many cycles, the friction coefficient of the coating is unstable, which is related to the uneven coating surface during to the transitional stage (1000–2000 s) [48]. Figure 10a also shows that the friction coefficient of the coating gradually decreases and finally stabilizes with the increasing number of wear cycles. The average friction coefficient of the composite coatings with 10, 15, 20 and 25 wt % Ni reaches approximately 0.5178, 0.5192, 0.5291, and 0.5088, respectively, which is consistent with the obtained surface roughness values. According to the wear weight loss calculation (Figure 10b), the relative wear resistance values of the composite coatings with 10, 15, 20 and 25 wt % Ni are 1.6 × 10^−3^, 3.1 × 10^−3^, 2.5 × 10^−3^ and 1.7 × 10^−3^ g, respectively. Compared with the weight loss of the pure Zn coating (0.0228 g), the weight loss of the (90 wt % Zn–10 wt % Ni)-30 vol % Al_2_O_3_ coating is approximately 14 times lower than the pure Zn coating. The (90 wt % Zn–10 wt % Ni)-30 vol % Al_2_O_3_ coating demonstrates the best wear resistance, mainly because wear resistance is proportional to the hardness of the material [49,50], which is consistent with the microhardness values. Considering the higher microhardness of Al_2_O_3_ and Ni compared to Zn, the microhardness of the Zn coating is increased by adding of Al_2_O_3_ and Ni (700 HV, 356 HV vs. 45 HV). Along with the results in Table 2, these results show that the Al_2_O_3_ content in (90 wt % Zn–10 wt % Ni)-30 vol % Al_2_O_3_ is twice that of the other coatings, and the Ni content in (90 wt % Zn–25 wt % Ni)-30 vol % Al_2_O_3_ is also higher than that of the other coatings. This further illustrates the roles of the hard Al_2_O_3_ phase and Ni in the wear resistance of the coatings.

**Figure 11:**
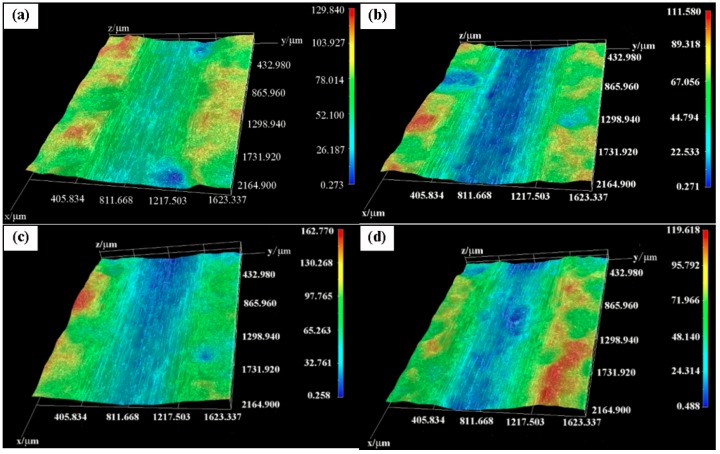
Three-dimensional morphology of the worn surfaces of the composite coatings with various Ni powder contents: (**a**) 10 wt %, (**b**) 15 wt %, (**c**) 20 wt % and (**d**) 25 wt %.

The three-dimensional morphology of the worn surface of the composite coatings is shown in Figure 11. It can be seen that the wear marks of (90 wt % Zn–10 wt % Ni)-30 vol % Al_2_O_3_ are shallower than those of the other three coatings, further confirming that this coating offers the best wear resistance. These results are in agreement with the wear loss values (Figure 10b).

**Figure 12:**
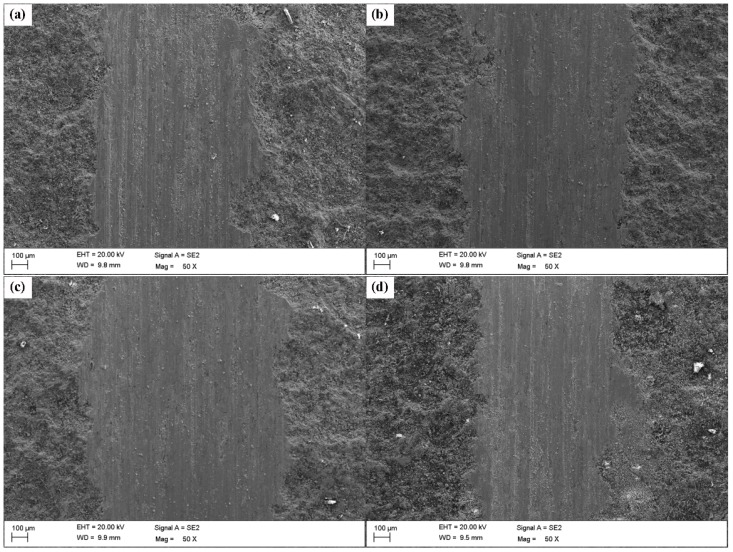
SEM images of the worn surfaces of the composite coatings with various Ni powder contents: (**a**) 10 wt %, (**b**) 15 wt %, (**c**) 20 wt % and (**d**) 25 wt %.

SEM images of the worn surfaces of the composite coatings with 10, 15, 20 and 25 wt % Ni are shown in Figure 12. Shallow scratches appear on the coating surface, and the direction of the furrows is the same as that of the sliding ball due to the relative sliding occurring between abrasive grains and the coating surface. When the coating surfaces are subjected to a normal load, severe deformation and adhesion occur on the coating surfaces, which are mainly due to the roughness and waviness of the contacting surface [51]. There is a small amount of scratching on the coating surface, mainly due to the relative sliding by the abrasive particles cutting from the adhesion points. Combined with the analysis of dry friction and wear behavior, the wear mechanism of the Zn-Ni-Al_2_O_3_ composite coatings can be divided into three stages. Initial wear regimes occur in the first few contact events between two surfaces that have not previously been in contact. At this stage, the COF of the coating rapidly increases, and few wear fragments are produced. Depending on the collisions between the chrome steel ball and Zn-Ni-Al_2_O_3_ coating, few wear fragments are produced, which then adhere mainly to the chrome steel surface forming layers, which is frequently called the layer formation regime. In the regimes of the layer formation, wear fragments gradually agglomerate on a stable layer on the chrome steel surface, and the frictional heating in the contact results in the oxidation of the layer and surface. With increasing wear repetitions, abrasive wear is caused by the layer segments and the abrasive particles cutting from the adhesion points. At this stage, the COF and wear gradually increase within creasing number of wear repetitions. In the regimes of steady-state wear, a stable layer is produced on the chrome steel surface, and the stable layer inhibits direct adhesion between the chrome steel and Zn-Ni-Al_2_O_3_ coating, which also explains the lower coefficient of friction and wear rate. Thus, the wear mechanism of Zn-Ni-Al_2_O_3_ composite coatings with various Ni powder fractions is abrasive and adhesive wear.

## 4. Conclusions

In the present study, Zn-Ni-Al_2_O_3_ composite coatings with different Ni contents were successfully fabricated on Q235 substrate by the LPCS technique, and their composition, microstructure, mechanical properties and tribological behavior were examined. The conclusions can be summarized as follows:

- According to the XRD, the composite coatings are primarily composed of metallic-phase Zn and Ni and ceramic-phase Al_2_O_3_. The cross-sectional morphology revealed thick, dense coatings with a wave-like stacking structure. The Al_2_O_3_ content of the composite coatings gradually decreases with increasing of Ni content.
- The deposition process of Zn, Ni and Al_2_O_3_ particles by the LPCS method was examined, and mechanical interlocking was found to be the deposition mechanism. When the Zn:Ni mass ratio of mixed powders was less than or equal to 1:4, the Ni mixed with Zn powders can be easily deposited.
- The bond strength and the microhardness of the coatings do not change significantly when the Ni content varies. The bond strength of the composite coatings is higher than 20 MPa and the cohesion of the coating is higher than that of the coating/matrix. The average microhardness of the coatings is above 120 HV_0.3_, which is 2.5 times that of the pure Zn coating.
- The average COF values of the composite coatings with 10, 15, 20 and 25 wt % Ni reach approximately 0.5178, 0.5192, 0.5291, and 0.5088, respectively. The wear resistance of the Zn-Ni-Al_2_O_3_ composite coatings is far superior to that of pure Zn coatings, especially for the (90 wt % Zn–10 wt % Ni)-30 vol % Al_2_O_3_ coating, which is primarily due to the presence of Al_2_O_3_ and Ni. SEM micrographs of the worn surfaces reveal that the wear mechanism of Zn-Ni-Al_2_O_3_ composite coatings is abrasive and adhesive wear.

## Figures and Tables

**Table 1:** LPCS processparameters of spraying powders.

Process Parameters	Unit	Value
Standoff distance	mm	25
Powder feed rate	g/min	12
Gas temperature	°C	400
Traverse speed	mm/s	20

**Table 2:** EDS analysis of LPCS Zn-Ni composite coatings.

Samples	Element Distribution (wt %)			
	O	Al	Ni	Zn
10 wt % Ni	10.09	11.72	14.01	64.18
15 wt % Ni	7.92	8.33	25.24	58.51
20 wt % Ni	5.33	6.14	33.88	54.65
25 wt % Ni	4.80	5.32	52.59	37.20
